# Loneliness Among Older Adults in Latin America, China, and India: Prevalence, Correlates and Association With Mortality

**DOI:** 10.3389/ijph.2021.604449

**Published:** 2021-03-31

**Authors:** Qian Gao, A. Matthew Prina, Martin Prince, Daisy Acosta, Ana Luisa Sosa, Mariella Guerra, Yueqin Huang, Ivonne Z. Jimenez-Velazquez, Juan J. Llibre Rodriguez, Aquiles Salas, Joseph D. Williams, Zhaorui Liu, Isaac Acosta Castillo, Rosie Mayston

**Affiliations:** ^1^ Health Service and Population Research, Institute of Psychiatry, Psychology and Neuroscience, King’s College London, London, United Kingdom; ^2^ Geriatric Section, Internal Medicine Department, Universidad Nacional Pedro Henriquez Ureña, Santo Domingo, Dominican Republic; ^3^ National Institute of Neurology and Neurosurgery of Mexico, National Autonomous University of Mexico, Mexico City, Mexico; ^4^ Instituto de la Memoria Depresion y Enfermedades de Riesgo, Lima, Peru; ^5^ Social Psychiatry and Behavioral Medicine, Institute of Mental Health, Peking University, Beijing, China; ^6^ Geriatrics Program, Internal Medicine Department, School of Medicine, Medical Sciences Campus, University of Puerto Rico, San Juan, Puerto Rico; ^7^ Facultad de Medicina Finlay-Albarran, Medical University of Havana, Havana, Cuba; ^8^ Medicine Department, Caracas University Hospital, Faculty of Medicine, Universidad Central de Venezuela, Caracas, Venezuela; ^9^ Department of Community Health, Voluntary Health Services, Chennai, India; ^10^ Global Health and Social Medicine, King’s Global Health Institute, Social Science and Public Policy, King’s College London, London, United Kingdom

**Keywords:** loneliness, mortality, older adults, low- and middle-income countries, social ageing

## Abstract

**Objectives:** This study was designed to explore prevalence and correlates of self-reported loneliness and to investigate whether loneliness predicts mortality among older adults (aged 65 or above) in Latin America, China and India.

**Methods:** The study investigated population-based cross-sectional (2003–2007) and longitudinal surveys (follow-up 2007–2010) from the 10/66 Dementia Research Group project. Poisson regression and Cox regression analyses were conducted to analyse correlates of loneliness and its association with mortality.

**Results:** The standardised prevalence of loneliness varied between 25.3 and 32.4% in Latin America and was 18.3% in India. China showed a low prevalence of loneliness (3.8%). In pooled meta-analyses, there was robust evidence to support an association between loneliness and mortality across Latin American countries (HR = 1.13, 95% CI 1.01–1.26, I^2^ = 10.1%) and China (HR = 1.58, 95% CI 1.03–2.41), but there were no associations in India.

**Conclusion:** Our findings suggest potential cultural variances may exist in the concept of loneliness in older age. The effect of loneliness upon mortality is consistent across different cultural settings excluding India. Loneliness should therefore be considered as a potential dimension of public health among older populations.

## Introduction

Loneliness has been described as the “unpleasant phenomenon stemming from the discrepancy between desired and achieved levels of social relations” (1). Evidence suggests loneliness may be a common experience among older people, with estimates ranging from 19.6 to 34.0% among people aged over 65 in Europe (2), and 25–29%, among participants aged 70+ years old in the United States (3). The frequency of loneliness in older age is consistent with the social effects of ageing: the accumulation of life events and processes that have the potential to destabilise social relations. For example, widowhood and onset of disability are more likely in older age (1) and personal and friendship networks decrease throughout adulthood (4). Loneliness is a subjective evaluation of an individual’s context which is made up of perceived deficits in social contacts and unmet social needs, which can be affected by both quantity and quality of personal relationships (5). Social isolation is a related concept which reflects an objective condition of lacking integration into social networks and social contacts, which is commonly associated with loneliness. However, the concepts are not interchangeable: it is not necessary to be socially isolated in order to experience loneliness (6).

Research carried out in high income country (HIC) settings has consistently identified sociodemographic correlates of loneliness: female gender (7, 8), older age (8), low educational level (9), poor income (10), being widowed (11, 12), living alone and low quality of social relationships (8, 11); as well as a number of adverse health outcomes, such as low well-being (12), mobility difficulties (12, 13), chronic diseases (14), cardiovascular ill-health (15, 16), depression and dementia (5). Direction of causality has been difficult to ascertain. This is due to the cross-sectional design of most studies as well as the possibility of reverse causality, which, due to the likely complexity of the relationships between factors such as depression, is often retained, even in the context of longitudinal designs (16). Evidence about the effect of loneliness on mortality in older adults is also mixed (16). Findings from studies carried out in Western Europe, United States and China suggest that loneliness has an independent effect upon mortality, with hazard ratios (HRs) ranging from 1.45 (95% CI 1.11–1.88) in the United States (17) to 1.17 (95% CI 1.02–1.33) in Finland (18).

Many of the factors thought to influence loneliness among older people (age, gender, widowhood, quality of social relationships) have been found to be relevant across different cultural settings (19). Qualitative findings from low- and middle-income countries (LMICs) have revealed similar narratives among older people: with change in family/social relationships like loss of spouses, being separated, reduced decision-making power and autonomy within the family, living alone and reduced social connections identified as the circumstances which shape experiences of loneliness (20–23). However, cultural differences in conceptions of the roles of older people, familial relationships and broader societal differences may potentially result in variation in experience and reporting of loneliness (24). Given the expected roles of culture and context in shaping experience of older age, findings about the effects of loneliness are not necessarily transferable across settings. We did not identify any studies which compared differences in loneliness prevalence and correlates across different cultural settings. Given the possible importance of loneliness as a potentially modifiable predictor of adverse outcomes including mortality (16, 18, 25), there is a need for research which examines loneliness in different cultural settings.

Using data from the 10/66 Dementia Research Group (10/66 DRG) study collected in Latin America, China and India, our analysis addresses three objectives related to gaining a better understanding of loneliness among older people in LMICs: 1) To estimate the prevalence of loneliness; 2) To examine whether correlates of loneliness identified from the literature were associated with the measure of loneliness used in the 10/66 DRG studies across different cultural settings; 3) To test the hypothesis that, after adjustment for sociodemographic and health-related correlates, loneliness was independently associated with mortality.

## Methods

### Context and Data Resources

The study is a secondary data analysis using population-based cross-sectional and longitudinal surveys from the 10/66 DRG project. The surveys were conducted among older adults (aged 65 or above) living in 12 geographically defined catchment areas in eight countries, including Cuba (Havana/Matanzas-urban), Dominican Republic (Santo Domingo-urban), Puerto Rico (Bayamon-urban), Venezuela (Caracas-urban), Peru (Lima-urban & Canete Province-rural), Mexico (Mexico City-urban and Morelos state-rural), China (Xicheng-urban and Daxing-rural) and India (Chennai-urban and Vellore-rural). Catchment areas were defined geographic areas selected for accessibility (26, 27). All assessments used in field work have been translated into relevant local languages (Ibero-American Spanish, Tamil and Mandarin). Baseline data were collected between 2003 and 2007, with high response rates for the baseline surveys across sites (72–98%). All participants were interviewed and assessed comprehensively, with interviews lasting around 2–3 h. For those lacking capacity of consent or with communication difficulties caused by dementia, mental or physical illnesses, informants were interviewed about the older person. The follow-up surveys were conducted between 2007 and 2010, which attempted to trace and re-interview all baseline study participants with at least three times visits were considered as untraced. Participants who moved outside the original catchment areas were re-interviewed in follow-up survey as well. Follow-up surveys were carried out in all baseline study sites except for the rural study site in India. The original study ethical approval was obtained from local ethical committees and the King’s College London Research Ethics Committee. A detailed protocol has been published elsewhere (28).

### Measures

#### Exposure

Information on self-reported experience of loneliness was assessed by a single item on the Geriatric Mental State (GMS)-Automated Geriatric Examination for Computer Assisted Taxonomy (AGECAT) package (29): “Do you feel lonely?,” with three response options (“no”/“yes but mild to moderate intensity, infrequent or fleeting”/“yes and severe, frequent or persistent”). The assessment of loneliness was coded as “feeling lonely” if the item was rated positive and recoded into a binary variable (yes/no). Single-item measures of loneliness have been commonly used in population-based studies across different cultural settings (30, 31).

#### Outcome

Vital status of older participants was ascertained during the initial “door-knocking” process, carried out with all households who participated in baseline surveys, to assess changes to households during the follow-up period. The starting of the risk time was dates of the baseline survey. The date of death, the date of follow-up for re-interviewed participants who moved away from the original catchment areas, or the median date of follow-up for participants who refused the interview was recorded and censored as survival time (28, 32).

#### Covariates

Socio-demographic information on age, gender, marital status, education, pension and wealth were assessed by a standard socioeconomic demographic questionnaire. Age was measured as a continuous variable, and recoded into four bands (65–69, 70–74, 75–79, 80+) for analyses; marital status categorised into four groups (never married, married/co-habiting, widowed, divorced/separated); level of education was classified as five groups (none, incomplete primary, completed primary, completed secondary (metric), and completed tertiary (college)/further education). Pension was a dichotomous variable, assessed by whether older people received any government or occupational pension; household assets index was recorded by summing up the number of household assets owned (i.e., car, television, refrigerator, telephone, mains electricity, mains water, plumbed toilet) and recoded into four quarters, which used to as an indicator for older people’s wealth. Social network types were operationally assessed and generated into Wenger’s network typology by using the Practitioner Assessment of Network Type (PANT), as a categorical variable including locally integrated, locally self-contained, wider community focused, family dependent and private network types. The locally integrated support network type represents the widest access to social support, whilst the private network type refers to the narrowest network type, which can be described as an absence of local or nearby family and friends and with low levels of community contact or involvement. The details of social network measurement, algorithm and explanation have been described elsewhere (33). Whether participants lived alone was a single item question (yes/no).

Physical impairments were assessed through a self-reported list of 11 common physical impairments (i.e., arthritis or rheumatism; eyesight problems; hearing difficulties or deafness; persistent cough; breathlessness, difficulty breathing or asthma; high blood pressure; heart trouble or angina; stomach or intestine problems; faints or blackouts; paralysis, weakness or loss of one leg or arm; skin disorders) and recoded and categorised as “no impairments,” “one to two impairments,” and “three or more impairments” (32). Care dependence was assessed by asking open-ended questions to the key informant about participant’s needs for care and then coded by interviewers and categorised as “required no care” or “care some” or “need much care”, which was used here to define “dependence” at both baseline and follow-up surveys (26). Depression was measured by structured clinical interview (the GMS), and derived from its computerised algorithm AGECAT, which provided International Classification of Diseases-10 (ICD-10) depressive episode diagnoses (mild/moderate/severe) (29, 34). Dementia was ascertained by either the cross-culturally validated 10/66 dementia diagnosis algorithm or DSM-IV (Diagnostic and Statistical Manual of Mental Disorders) dementia diagnostic criterion (35–37).

### Statistical Analysis

All analyses were conducted using STATA version 15 (StataCorp. 2017. Stata Statistical Software: Release 15. College Station, TX: StataCorp LLC.). Initial analyses were presented on the prevalence and correlates of self-reported loneliness in the baseline dataset. 346 participants who did not respond to the loneliness question were excluded from our analyses (Cuba *n* = 47; Dominican Republic *n* = 11; Peru *n* = 49; Venezuela *n* = 21; Mexico *n* = 11; Puerto Rico n = 95; China *n* = 61; India *n* = 51). No follow-up survey was conducted in the rural site of India hence participants from this site were excluded in the mortality analyses. A weighting variable was provided by the 10/66 DRG baseline dataset for each country for direct standardisation, with the whole sample as the standard population. The crude and direct standardised prevalence of loneliness adjusted for age, gender and education with robust 95% confidence intervals (CI) was estimated by accounting for household clustering across the whole sample as well as within each country. We used Poisson regression models to examine all hypothesised and theory-driven correlates of loneliness in each country, by adjusting for age, gender, education, household assets, pension, marital status, social network, living alone, physical impairments, care dependence, depression and dementia. The adjusted prevalence ratios (PRs) (with robust 95% CI) for loneliness were reported separately for each country, and then pooled with fixed effect meta-analysis across all study sites combined and Latin American countries.

Kaplan-Meier curves were generated to compare crude mortality between participants with and without loneliness. The differences between the survival curves were tested using Log-rank tests. Univariate and Multivariable Cox proportional hazards regression models were conducted to examine the association between loneliness and mortality, while the Schoenfeld residuals test was used to test the proportional hazard assumptions. Multivariable Cox models were built based on a combination of literature review, results from bivariate analysis and consideration of distribution of characteristics. Variables were gradually added in blocks. Blocks were determined from background literature, based on knowledge of factors associated with loneliness (sociodemographic, social isolation, physical health, mental health). Variables were selected for inclusion in blocks if considered as potential confounders on the basis of being correlated with loneliness in bivariate analyses. Three sets of models were built: for Latin American countries, India and for China. Across six Latin American countries and India, models were ultimately adjusted for (Model I) baseline socio-demographic factors (age, gender, education and household assets), then (Model II) adding social network, followed by (Model III) adding dependence; finally (Final Model) depression and dementia were included. Considering building the model for China, previous 10/66 DRG studies based on the same samples showed that there was no association between social network and mortality in the adjusted model for China. As only family dependent social network type showed an association in the unadjusted model in for the Chinese 10/66 baseline dataset (33), and living alone played a role in reflecting family connections and marital status, we decided to include living alone instead of social network in Multivariable Cox model building for China in this study. Finally, we introduced time-varying interactions for age and education for fitting our Cox models for China. The likelihood ratio test was used to test the fit of each model. The adjusted HRs for loneliness were reported and fitted in each site separately and then combined with fixed effect meta-analysis to generate pooled HRs across Latin American countries. Higgins I^2^ was measured to estimate the level of heterogeneity across the estimates in different settings. Lower than 40% heterogeneity was considered as negligible, and 40–60% was regarded as moderate heterogeneity (38).

## Results

### Baseline Characteristics of Study Samples and Vital Status at Follow Up

A total of 16,685 older adults (65 years and above) were included in the baseline sample across eight LMICs. Sample numbers were around 2000 (ranging from 2,897 to 1,884) in each study country except Cuba which had a slightly larger sample (*n* = 2,897). The mean age of participants was 74.1 (6.9) years and evenly distributed across the five bands, except that in three sites (Venezuela, China and India), there were proportionately fewer in the oldest age category. Overall, 62.4% of samples were female. Across countries the female proportion exceeded the male but was distributed evenly (Supplementary File S1). 13,673 (86.9%) of 15,733 participants interviewed at baseline participated in follow-up (re-interviewed or vital status ascertained). There were some differences in the loss to follow-up rates by baseline loneliness status in study countries (14.6% for loneliness vs. 12.6% without loneliness), especially for Cuba, Venezuela and China (*p* < 0.05). 2,439 participants were recorded dead, which accounted for 17.8% of those successfully traced. Overall, median follow-up years was 4.0 years (3.0–4.9), equivalent to a total of 53,139.4 person years of follow-up. The proportion of baseline participants where vital status was ascertained was evenly distributed across countries. Mortality was highest in Dominican Republic (27.1%), followed by China, Cuba and India, which were higher than other countries. Differences in timing of data collection in each country led to a variance on follow-up years, with median follow-up years shorter in India, Mexico and Peru (Table 1).

**TABLE 1:** Characteristics of vital status at follow up for those whose vital status was known (The 10/66 Dementia Research Group study 2003–2010).

Characteristics	Cuba	Dominican Republic	Peru	Venezuela	Mexico	Puerto Rico	China	India	Overall
Cohort at baseline	2,897	2,000	1,884	1,944	1,992	1914	2,101	1,001	15,733
Vital status ascertained at follow-up (N, % of baseline sample)	2,590 (89.4%)	1,696 (84.8%)	1707 (90.6%)	1,679 (86.4%)	1833 (92.0%)	1,492 (78.0%)	1931 (91.9%)	745 (74.4%)	13,673 (86.9%)
Lose to follow-up (N, % of participants reporting loneliness at baseline)^a^	64 (8.4%)	109 (17.1%)	55 (9.8%)	80 (16.6%)	54 (7.8%)	139 (24.3%)	15 (24.6%)	62 (30.5%)	578 (14.6%)
Lose to follow-up (N, % of participants reporting no loneliness at baseline)	243 (11.4%)	195 (14.3%)	122 (9.2%)	185 (12.6%)	105 (8.1%)	283 (21.1%)	155 (7.6%)	194 (24.3%)	1,482 (12.6%)
Deaths (N, % of those with vital status determined)	576 (22.2%)	459 (27.1%)	131 (7.7%)	187 (11.1%)	205 (11.2%)	252 (16.9%)	478 (24.8%)	151 (20.3%)	2,439 (17.8%)
Median length of follow-up (years; IQR)	4.3 (3.6–5.0)	5.0 (3.7–5.1)	3.1 (2.6–3.7)	4.2 (4.0–4.8)	3.0 (2.9–3.2)	4.3 (3.8–4.7)	4.9 (4.5–5.3)	2.9 (2.5–3.6)	4.0 (3.0–4.9)
Person years of follow-up	10,729.3	7,422.6	5,253.3	6,986.9	5,335.4	6,240.6	8,980.4	2,190.8	53,139.4

^a^ Loss to follow-up across loneliness are statistically different in Cuba, Venezuela, China and overall (*p* < 0.05).

### Prevalence of Loneliness and Its Correlates

The standardised prevalence of loneliness varied between 25.3 and 32.4% by Latin American countries. The highest standardised prevalence of loneliness was 32.4% (95%CI 29.9–34.8%) in Mexico, followed by Puerto Rico 32.2% (95% CI 29.0–35.4%). The standardised prevalence of loneliness was lower in India (18.3%, 95% CI 16.0–20.6%) compared to Latin American countries, whilst China showed an extremely low prevalence (3.8%, 95% CI 2.6–4.9%) (Table 2). According to the results of Multivariable Poisson regression, pooled adjusted PR for loneliness showed that, across all countries, loneliness was significantly associated with female gender, lower education level, lower household assets index, being widowed (pooled PR = 1.31, 95% CI 1.17–1.46, I^2^ = 36.5%) or divorced/separated (pooled PR = 1.27, 95% CI 1.13–1.43, I^2^ = 0%), with a narrower social network type, living alone (pooled PR = 1.42, 95% CI 1.32–1.52, I^2^ = 51.5%), with more physical impairments, with care dependence, depression and dementia. Compared to people with locally integrated social network, participants with private social network had a significant association with loneliness (pooled PR = 1.14, 95% CI 1.03–1.27, I^2^ = 43.4% all countries combined; pooled PR = 1.20, 95% CI 1.07–1.35, I^2^ = 38.2%, Latin American countries combined). Pooled PR for more physical impairments was 1.32 (95% CI 1.27–1.37, I^2^ = 32.8%, all countries combined). Older people with care dependence were correlated with feeling lonely (pooled PR = 1.14, 95% CI 1.02–1.27, I^2^ = 0% all countries combined; pooled PR = 1.13, 95% CI 1.0–1.26, I^2^ = 16.0%, Latin American countries combined). Depression showed a significant pooled effect on loneliness (pooled PR = 1.95, 95% CI 1.83–2.07, I^2^ = 73.2% across all countries; pooled PR = 1.92, 95% CI 1.79–2.06, I^2^ = 0%, Latin American countries combined) (Table 3). The adjusted PRs for loneliness were reported separately for study site in Supplementary File S2.

**TABLE 2:** Crude and standardised prevalence of self-reported loneliness in Latin America, China, and India (The 10/66 Dementia Research Group study 2003–2007).

Sites	Loneliness/baseline sample	Crude prevalence (95% CI)^a^	Standardised prevalence (95%CI)^b^	Standardised prevalence (95%CI)^c^
Cuba	758/2,897	26.2% (24.6–27.8%)	25.8% (24.2–27.4%)	27.9% (25.5–30.3%)
Dominican Republic	638/2,000	31.9% (29.8–34.0%)	31.2% (29.1–33.2%)	30.0% (27.7–32.4%)
Peru	561/1,884	29.8% (27.7–31.9%)	29.9% (27.9–32.0%)	29.5% (27.1–32.0%)
Venezuela	481/1,944	24.7% (22.8–26.8%)	24.8% (22.8–26.7%)	25.3% (22.8–27.9%)
Mexico	695/1,992	34.9% (32.8–37.0%)	34.4% (32.3–36.4%)	32.4% (29.9–34.8%)
Puerto Rico	573/1,914	29.9% (27.9–32.1%)	29.3% (27.2–31.4%)	32.2% (29.0–35.4%)
China	61/2,101	2.9% (2.2–3.7%)	3.2% (2.4–4.0%)	3.8% (2.6–4.9%)
India	203/1,953	26.9% (24.9–29.0%)	27.8% (25.6–29.9%)	18.3% (16.0–20.6%)

^a^ CI, confidence intervals.

^b^ Adjusted for age and gender.

^c^ Adjusted for age, gender and education.

**TABLE 3:** Correlates of loneliness with adjusted prevalence ratios (robust 95% confidence interval) in Latin America, China, and India (The 10/66 Dementia Research Group study 2003–2007).

Characteristics	Cuba	Dominican Republic	Peru	Venezuela	Mexico	Puerto Rico	China	India	All countries *Pooled PR Estimate*	Higgins I^2^ (%)	Latin American countries^a^ *Pooled PR Estimate*	Higgins I^2^ (%)
Age (65–110 years old)	0.99 (0.98–1.00)	1.00 (1.00–1.01)	0.99 (0.98–1.00)	0.99 (0.98–1.00)	1.01 (1.00–1.02)	0.98 (0.97–0.99)	1.00 (0.95–1.04)	1.00 (0.99–1.02)	1.0 (0.99–1.0)	71.1	1.0 (0.99–1.0)	79.1
Gender male (ref. female)	0.77 (0.64–0.92)	1.02 (0.88–1.19)	0.73 (0.62–0.86)	0.84 (0.69–1.03)	0.82 (0.71–0.94)	0.76 (0.64–0.91)	1.12 (0.68–1.86)	0.96 (0.79–1.16)	0.84 (0.79–0.90)	51.8	0.83 (0.77–0.88)	55.2
Higher education (ref. lower)	0.98 (0.91–1.04)	1.00 (0.93–1.07)	1.01 (0.94–1.07)	1.01 (0.92–1.10)	0.90 (0.84–0.96)	0.96 (0.90–1.02)	0.97 (0.78–1.21)	0.76 (0.69–0.85)	0.96 (0.93–0.98)	75.1	0.97 (0.95–1.0)	37.5
Household assets (ref. fewer)	0.96 (0.89–1.03)	0.89 (0.83–0.95)	0.91 (0.84–0.98)	0.93 (0.85–1.02)	0.89 (0.84–0.94)	1.01 (0.93–1.09)	1.15 (0.88–1.49)	0.81 (0.75–0.87)	0.91 (0.89–0.94)	68.8	0.92 (0.90–0.95)	44.1
Pension (ref. none)	0.86 (0.72–1.02)	0.92 (0.80–1.06)	0.93 (0.80–1.06)	0.92 (0.79–1.08)	0.85 (0.76–0.95)	1.12 (0.98–1.28)	2.25 (0.73–6.98)	0.97 (0.82–1.14)	0.94 (0.89–0.99)	47.5	0.93 (0.89–0.98)	53.5
Marital status (ref. never married)												
Married/cohabiting	0.68 (0.53–0.88)	0.81 (0.59–1.11)	1.01 (0.78–1.31)	0.75 (0.55–1.02)	0.82 (0.62–1.08)	0.86 (0.62–1.18)	N/A^b^	0.57 (0.31–1.06)	0.81 (0.72–0.90)	3.8	0.82 (0.73–0.92)	0.0
Widowed	1.18 (0.93–1.50)	1.30 (0.97–1.74)	1.50 (1.16–1.94)	1.52 (1.15–2.01)	1.17 (0.89–1.53)	1.46 (1.06–2.00)	N/A^b^	0.62 (0.33–1.16)	1.31 (1.17–1.46)	36.5	1.34 (1.12–1.49)	0.0
Divorced/separated	1.25 (0.98–1.61)	1.33 (1.00–1.78)	1.49 (1.06–2.08)	1.18 (0.86–1.61)	1.24 (0.91–1.68)	1.34 (0.97–1.87)	N/A^b^	0.72 (0.36–1.44)	1.27 (1.13–1.43)	0.0	1.29 (1.14–1.46)	0.0
Social network (ref. locally integrated)												
Locally self-contained	1.15 (0.88–1.50)	1.29 (1.04–1.59)	1.37 (1.05–1.78)	1.30 (1.01–1.67)	1.30 (1.03–1.64)	1.29 (1.06–1.57)	1.35 (0.37–4.99)	0.96 (0.69–1.34)	1.26 (1.15–1.38)	0.0	1.28 (1.17–1.41)	0.0
Wider community-focused	1.27 (0.89–1.81)	1.21 (0.99–1.48)	1.39 (1.06–1.82)	1.11 (0.87–1.41)	0.87 (0.62–1.22)	1.34 (1.09–1.66)	5.06 (0.57–44.91)	0.64 (0.27–1.52)	1.21 (1.09–1.34)	29.0	1.21 (1.10–1.34)	18.4
Family dependent	0.97 (0.80–1.18)	0.96 (0.81–1.13)	1.17 (1.00–1.37)	1.14 (0.94–1.38)	0.96 (0.85–1.09)	1.13 (0.93–1.37)	0.71 (0.20–2.52)	1.27 (1.07–1.51)	1.07 (1.0–1.13)	40.1	1.04 (0.97–1.11)	25.4
Private	1.26 (1.00–1.59)	1.22 (0.95–1.57)	0.98 (0.64–1.53)	0.68 (0.43–1.07)	1.33 (0.98–1.80)	1.33 (1.04–1.70)	1.66 (0.46–5.96)	0.93 (0.74–1.16)	1.14 (1.03–1.27)	43.4	1.20 (1.07–1.35)	38.2
Living alone (ref. living with others)	1.62 (1.35–1.94)	1.35 (1.14–1.59)	1.23 (0.95–1.59)	0.98 (0.68–1.42)	1.27 (1.09–1.48)	1.47 (1.23–1.74)	1.02 (0.35–3.00)	1.72 (1.44–2.06)	1.42 (1.32–1.52)	51.5	1.37 (1.26–1.48)	42.8
Physical impairments (ref. less)	1.41 (1.27–1.55)	1.26 (1.15–1.39)	1.39 (1.27–1.53)	1.27 (1.15–1.41)	1.27 (1.18–1.38)	1.39 (1.27–1.53)	1.83 (1.24–2.70)	1.25 (1.14–1.38)	1.32 (1.27–1.37)	32.8	1.33 (1.28–1.38)	21.9
Dependence (ref. no needs for much care)	1.01 (0.73–1.39)	1.26 (1.03–1.53)	0.83 (0.55–1.26)	1.28 (0.92–1.78)	0.97 (0.75–1.25)	1.21 (0.94–1.57)	0.99 (0.39–2.52)	1.43 (0.89–2.31)	1.14 (1.02–1.27)	0.0	1.13 (1.0–1.26)	16.0
Depression (ref. non-case)	2.29 (1.91–2.74)	1.87 (1.63–2.14)	1.90 (1.61–2.24)	1.84 (1.49–2.26)	1.85 (1.60–2.12)	1.86 (1.51–2.30)	11.67 (5.49–24.83)	1.96 (1.68–2.28)	1.95 (1.83–2.07)	73.2	1.92 (1.79–2.06)	0.0
Dementia (ref. non-case)	0.99 (0.77–1.28)	1.01 (0.83–1.23)	0.86 (0.63–1.17)	1.30 (1.00–1.68)	1.20 (1.01–1.41)	1.15 (0.90–1.47)	3.29 (1.41–7.70)	0.91 (0.72–1.13)	1.08 (1.0–1.18)	55.0	1.10 (1.0–1.21)	24.0

^a^ Latin American countries: Cuba; Dominican Republic; Peru; Venezuela; Mexico; Puerto Rico.

^b^ Estimates could not be obtained due to too few exposed in never married/divorced/separated categories for China.

### Loneliness and Mortality

**FIGURE 1:**
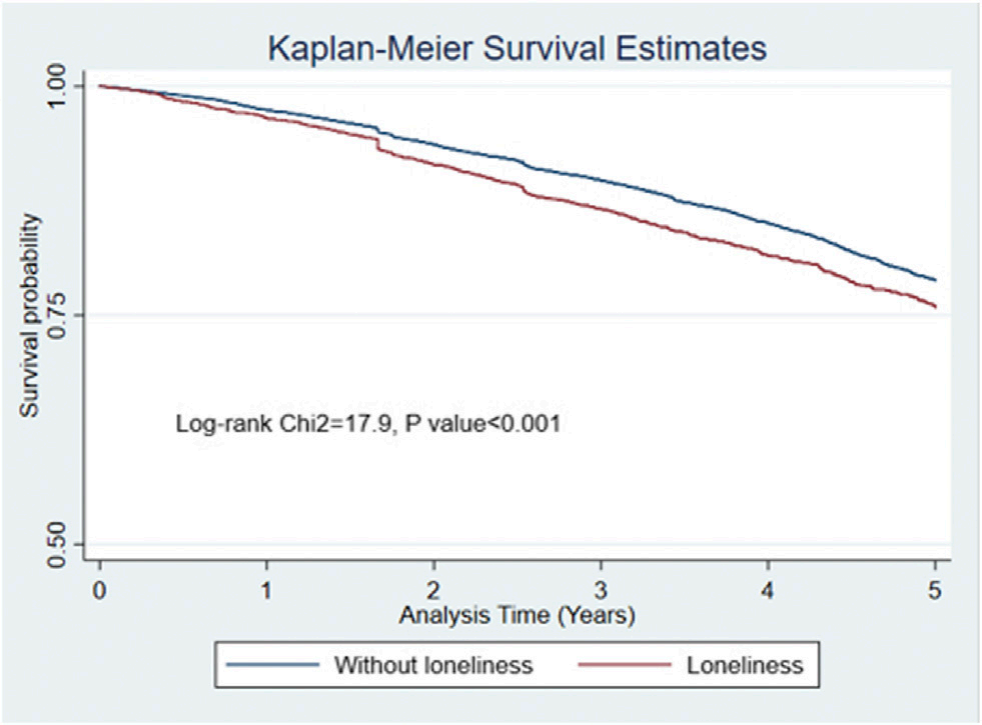
Kaplan-Meier survival curve for 5-year all-cause mortality, stratified by self-reported loneliness (The 10/66 Dementia Research Group study 2003-2010).

Crude Kaplan-Meier curves showed that participants with loneliness at baseline had lower 5-year survival rates (Figure 1), and with no difference between both gender and age strata (Supplementary File S3). Kaplan-Meier curves were plotted for the original three responses measure of loneliness, which showed a potential dose-response effect of increasing loneliness status (Supplementary File S4). Log-rank test gave *p* < 0.05 in all curves. Results of multivariable Cox models for Latin American countries, China and India showed that when restricted to Latin American countries pooled, the crude HR for loneliness was 1.25 (95% CI 1.14–1.38), with a moderate level of heterogeneity (I^2^ = 43.9%). In the final pooled analysis, after adjustment for all potential confounders, there was evidence for an association between loneliness and mortality (pooled adjusted HR = 1.13, 95% CI 1.01–1.26, I^2^ = 10.1%). In India, there was no association between loneliness and mortality across all models. However, in China, the effect of loneliness on mortality risk remained strong in the final model after adjusting for all confounders (adjusted HR = 1.58, 95% CI 1.03–2.41) (Table 4). Schoenfeld residuals test, *p* > 0.05 for all Cox models in Table 4.

**TABLE 4:** Meta-analysed pooled effect sizes for the association between loneliness and mortality in Latin America, China, and India (The 10/66 Dementia Research Group study 2003–2010).

Sites	Crude HR for loneliness	Model I^a^	Model II^b^	Model III^c^	Final model^d^
Cuba	1.21 (1.02–1.44)	1.25 (1.05–1.50)	1.24 (1.03–1.48)	1.24 (1.02–1.51)	1.19 (0.97–1.46)
Dominican Republic	1.39 (1.15–1.68)	1.24 (1.02–1.51)	1.24 (1.02–1.51)	1.14 (0.93–1.39)	1.13 (0.92–1.39)
Peru	0.76 (0.51–1.14)	0.82 (0.55–1.22)	0.80 (0.54–1.20)	0.77 (0.51–1.16)	0.74 (0.49–1.11)
Venezuela	1.51 (1.11–2.06)	1.64 (1.19–2.26)	1.56 (1.13–2.16)	1.50 (1.08–2.08)	1.37 (0.96–1.94)
Mexico	1.28 (0.98–1.69)	1.14 (0.86–1.51)	1.16 (0.88–1.54)	1.13 (0.85–1.51)	1.11 (0.82–1.49)
Puerto Rico	1.17 (0.89–1.52)	1.22 (0.93–1.60)	1.18 (0.90–1.55)	1.09 (0.82–1.45)	1.10 (0.83–1.46)
India	1.03 (0.69–1.56)	1.04 (0.67–1.62)	1.03 (0.66–1.61)	1.03 (0.66–1.61)	1.03 (0.65–1.62)
China	2.15 (1.43–3.25)	2.02 (1.40–2.91)	2.02 (1.40–2.92)^e^	1.85 (1.27–2.70)	1.58 (1.03–2.41)
Latin American countries^f^					
Pooled HR (95%CI)^g^	1.25 (1.14–1.38)	1.23 (1.11–1.36)	1.21 (1.10–1.34)	1.16 (1.05–1.29)	1.13 (1.01–1.26)
Higgins I^2^	43.9%	32.4%	25.8%	27.2%	10.1%

^a^ Model I: adjusted for age, gender, education and household assets.

^b^ Model II (all countries except China): adjusted for all variables in Model I plus social network.

^c^ Model III: adjusted for all variables in Model II plus dependence.

^d^ Final model: adjusted for all variables in Model III plus dementia and depression.

^e^ Model II (for China): adjusted for all variables in Model I plus living alone.

^f^ Latin American countries: Cuba; Dominican Republic; Peru; Venezuela; Mexico; Puerto Rico.

^g^ Hazard ratios (HR) and 95% confidence intervals (CI) are presented in the table.

## Discussion

The study found that the age, gender and education- standardised prevalence of loneliness ranged from 25.3% to 32.4% across Latin American countries, consistent with findings from the European Social Survey (2006–2007), which reported similar prevalence estimates of loneliness using a single-item measure with prevalence estimates ranging between 19.6% and 34.0% for older people aged 60 years old and above (2). In our study, we found a much lower standardised prevalence of loneliness in China of 3.8% compared to the 29.6% prevalence among older adults identified by a Chinese national ageing survey in 2000 (39). Although it is possible that the difference between our results and the nationally representative survey may reflect a real difference in levels of loneliness in the 10/66 DRG catchments, this result may be an artefact of our single item approach to measurement, a limitation which we discuss in more detail below. Our study showed that the direct standardised prevalence of loneliness was 18.3% in India, but there was scant previous evidence about the prevalence of loneliness among Indian elderly. The prevalence estimates of loneliness in this study showed a more similar pattern across Latin American countries, whereas lower prevalence was observed in China and India. Similar differences on prevalence of anxiety (40) and amnestic mild cognitive impairment (41) were observed in previous 10/66 DRG findings, where both studies reported a low prevalence in China. As applying a cross-cultural approach through using the same study design, sampling and measurements in this study, the inconsistence of prevalence estimates on the subjective feeling of loneliness across study countries is likely due to the measure of self-reported loneliness itself, as cultural variances may influence the conception of loneliness and stigma (41).

The hypothesis that loneliness increased mortality was supported by our results and was consistent across all countries except India. In Latin American countries, we found that after adjustment for all the sociodemographic and health-related confounders, pooled estimates still suggested robust evidence on an association between loneliness and mortality, consistent with the results of previous meta-analyses (42, 43). Whilst depression is undoubtedly associated with loneliness and vice versa (44), there is evidence to suggest that both depression and loneliness have independent effects on mortality despite common co-existence. In our study, adding depression and dementia to the model made very little difference to our results, thereby suggesting an independent association of loneliness to mortality. Despite a low prevalence, we found a strong association between loneliness and mortality in China, which was retained after adjustment for the same sets of characteristics. This might reveal the possibility that our measurement captured more intense cases of loneliness, but this remains untested. Both direct and indirect pathways have been posited as explanations for associations between loneliness and mortality in previous research. Indirect mechanisms include circular relationships between loneliness and behaviours (smoking, physical inactivity, poorer sleep) (44, 45) associated with poor health and their subsequent effects upon physiological outcomes; whilst direct mechanisms include emerging evidence to suggest that levels of social support may be linked to immune-mediated inflammatory processes (46).

Our findings regarding correlates were generally consistent with the literature from studies carried out in HIC settings. Older people, being female, living without a spouse, living alone, and those with lower socioeconomic status were more likely to be lonely. These factors often cluster together within individuals, are interrelated (e.g., form a “vicious cycle”) and are associated with low mood, a sense of meaninglessness or hopelessness as well as loneliness (20, 47). Locally integrated networks were most protective in terms of loneliness, whilst being dependent on others, having physical impairments, dementia and depression were all associated with loneliness. The results of qualitative work carried out in 10/66 sites and elsewhere offer further insight into the context in which loneliness in older age might emerge. Consistent with our current findings, a recent systematic review of qualitative studies from LMICs showed that loneliness was often characterised by older people as loss: of physical functioning, independence, close confidantes, social participation and sense of belonging within families and societies (20, 22, 23). Results from the 10/66 DRG INDEP study and a recent study in Ghana carried out among dependent older people suggest that changing societal expectations (greater female participation in paid employment, longer periods of education for young people) were putting a strain on social norms of intergenerational reciprocity (48, 49).

A key potential limitation of our study was that the measure of loneliness in this study was based on self-report of a single item. Although single-item measures of loneliness are commonly used, the validity of this approach has been questioned (3, 50). Compared to multi-item scales which capture variations in frequency and intensity of loneliness across different dimensions of the phenomenon, single-item measures risk under-reporting through simplification of the construct. This limitation might perhaps be considered particularly salient in cultural settings where loneliness might be associated with stigma and shame (22). In addition, a single item is unlikely to detect culturally mediated expressions of loneliness which may be conceptually linked but not recognised or labelled as “loneliness” by study participants. Nonetheless, the broad consistency of our findings with the literature regarding demographic, social and health factors associated with loneliness is reassuring, providing evidence to support concurrent validity of the measure. The reason for a single item measure detecting such a low prevalence of loneliness among our China sample remains unexplained and warrants further investigation. Given the low attrition overall, whilst we acknowledge the presence of differential loss to follow-up, we estimate the overall impact on our final results to be minimal. Our modelling strategy was guided by evidence developed from review of the literature. Constrained by secondary data analysis and available data, we cannot rule out the possibility that the association between mortality and loneliness was explained by unmeasured confounders. Although marital status, living alone and social network (to represent social relationships) were included in the analysis, it is unclear to what extent these variables capture the social reality of participants. Finally, in this cross cultural and population-based study, it is hard to eliminate missing data and loss to follow up, which might also lead to some limited selection bias as well as limiting the generalisability of the results to some extent.

The results of our study suggest that as is now the case in HICs, loneliness in LMICs among older populations should be considered a potential public health concern. We have demonstrated a significant minority of older people experience loneliness across diverse settings and that this construct has the expected associations with other demographic, social and health-related characteristics. We have shown that loneliness has a consistent effect upon mortality, independent of the effects of sociodemographic background, social network and mental health. Further research will be needed to understand the relationship between loneliness and health among older people in LMICs. Our results highlight the importance of considering social reality in the design of interventions designed to improve the health of older people. Although instrumental support and policies designed to facilitate this are undoubtedly needed, interventions targeting health outcomes will be missing opportunities to improve the lives of older people if they don’t consider the social dimensions of ageing, such as loneliness (24, 51).

## Data Availability

Publicly available datasets were analyzed in this study. This data can be found here: the 10/66 Dementia Research Group (https://1066.alz.co.uk) established an anonymised data sharing archive with monitored public access.
