# Supplementary material for: Loneliness Among Older Adults in Latin America, China, and India: Prevalence, Correlates and Association With Mortality
Source: Int J Public Health. 2021 Mar 31;66:604449. doi: 10.3389/ijph.2021.604449 (PMC8565277; doi:10.3389/ijph.2021.604449)
Supplement: Supplementary file 1 [file Table1.docx]

**Supplementary File 1 Demographic and socioeconomic characteristics by study countries (2003-2007)**

| **Characteristics** | **Cuba** | **Dominican Republic** | **Peru** | | **Venezuela** | **Mexico** | **Puerto Rico** | **China** | **India** | **All sites combined** |
| --- | --- | --- | --- | --- | --- | --- | --- | --- | --- | --- |
| **Numbers** | **(N=2897)** | **(N=2000)** | **(N=1884)** | | **(N=1944)** | **(N=1992)** | **(N=1914)** | **(N=2101)** | **(N=1953)** | **(N=16685)** |
| **Age (years) Mean (SD)** | 75.0 (7.0) | 75.2 (7.5) | 74.6 (7.2) | | 72.4 (6.8) | 74.3(6.6) | 75.9 (7.0) | 73.1 (6.0) | 71.9 (6.0) | 74.1 (6.9) |
| **Age - MV**^*^ | 6 | 0 | 0 | | 3 | 1 | 0 | 0 | 4 | 14 |
| 65-69 | 758 (26.2) | 531 (26.6) | 551 (29.0) | | 834 (43.0) | 541 (27.2) | 409 (21.4) | 692 (32.9) | 734 (37.7) | 5050 (30.3) |
| 70-74 | 779 (27.0) | 519 (26.0) | 489 (26.0) | | 467 (24.0) | 579 (29.0) | 447 (23.3) | 648 (30.8) | 652 (33.5) | 4580 (27.5) |
| 75-79 | 632 (21.9) | 396 (19.8) | 393 (20.9) | | 340 (17.5) | 424 (21.3) | 469 (24.5) | 438 (20.9) | 313 (16.0) | 3405 (20.4) |
| ≥80 | 722 (24.9) | 554 (27.7) | 451 (23.9) | | 300 (15.5) | 447 (22.5) | 589 (30.8) | 323 (15.4) | 250 (12.8) | 3636 (21.8) |
| **Female - MV** | 0 | 2 | 0 | | 0 | 0 | 5 | 0 | 14 | 21 |
| Female | 1879 (64.9) | 1316 (65.9) | 1148 (60.9) | | 1240 (63.8) | 1261(63.3) | 1285 (67.3) | 1186 (56.5) | 1085 (56.0) | 10400 (62.4) |
| Male | 1018 (35.1) | 682 (34.1) | 736 (39.1) | | 704 (36.2) | 731(36.7) | 624 (32.7) | 915 (43.5) | 854 (44.0) | 6264 (37.6) |
| **Education - MV** | 7 | 17 | 15 | | 30 | 2 | 7 | 0 | 1 | 79 |
| None | 73 (2.5) | 388 (19.6) | 116 (6.2) | | 152 (7.9) | 548 (27.5) | 58 (3.0) | 778 (37.0) | 1048 (53.7) | 3161 (19.0) |
| Incomplete primary | 642 (22.2) | 1018 (51.3) | 225 (12.0) | | 442 (23.1) | 861 (43.3) | 352 (18.5) | 261 (12.4) | 425 (21.8) | 4226 (25.5) |
| Completed primary | 965 (33.4) | 369 (18.6) | 708 (37.9) | | 963 (50.3) | 349 (17.5) | 399 (20.9) | 549 (26.1) | 324 (16.6) | 4626 (27.9) |
| Completed secondary | 716 (24.8) | 135 (6.8) | 506 (27.1) | | 265 (13.9) | 124 (6.2) | 695 (36.4) | 373 (17.8) | 112 (5.7) | 2926 (17.6) |
| Completed tertiary | 494 (17.1) | 73 (3.7) | 314 (16.8) | | 92 (4.8) | 108 (5.5) | 403 (21.1) | 140 (6.7) | 43 (2.2) | 1667 (10.0) |
| **Marital Status** - **MV** | 8 | 15 | 9 | | 35 | 1 | 5 | 0 | 2 | 75 |
| Never married | 268 (9.3) | 139 (7.0) | 204 (10.9) | | 187 (9.8) | 103 (5.2) | 113 (5.9) | 25 (1.2) | 25 (1.3) | 1064 (6.4) |
| Married/ Cohabiting | 1254 (43.4) | 585 (29.5) | 1074 (57.3) | | 918 (48.1) | 1005 (50.5) | 931 (48.8) | 1377 (65.5) | 983 (50.4) | 8127 (48.9) |
| Widowed | 913 (31.6) | 799 (40.2) | 506 (27.0) | | 545 (28.6) | 760 (38.2) | 634 (33.2) | 696 (33.1) | 898 (46.0) | 5751 (34.6) |
| \| Divorced/ Separated \| 454 (15.7) \| 462 (23.3) \| 91(4.8) \| 259 (13.6) \| 123 (6.1) \| 231 (12.1) \| 3 (0.2) \| 45 (2.3) \| 1668 (10.1) \| \| --- \| --- \| --- \| --- \| --- \| --- \| --- \| --- \| --- \| --- \|   ***Continued in next page*** | | | | | | | | | | |
| ***Supplementary File 1 Continued*** | | | | | | | | | | |
| **Household Assets - MV** | 7 | 5 | 0 | 0 | | 0 | 0 | 1 | 4 | 17 |
| **Mean (SD)** | 5.7 (1.0) | 5.0 (1.4) | 5.7 (1.1) | 6.2 (1.0) | | 5.1 (1.8) | 6.7 (0.6) | 5.6 (1.1) | 3.5 (1.7) | 5.4 (1.5) |
| **Any Pension (Yes)** | 2380 (82.2) | 608 (30.4) | 1234 (65.5) | 1143 (58.8) | | 979 (49.2) | 1019 (53.2) | 1056 (50.3) | 449 (23.0) | 8868 (53.2) |
| **Social network - MV** | 7 | 2 | 4 | 24 | | 0 | 5 | 0 | 0 | 42 |
| Locally integrated | 1992 (68.9) | 1171 (58.6) | 1094 (58.2) | 1071 (55.8) | | 906 (45.5) | 1161 (60.8) | 935 (44.5) | 1132 (57.9) | 9462 (56.9) |
| Locally self-contained | 157 (5.4) | 113 (5.7) | 106 (5.6) | 165 (8.6) | | 92 (4.6) | 213 (11.2) | 231 (11.0) | 91 (4.7) | 1168 (7.0) |
| Wider community-focused | 86 (3.0) | 203 (10.1) | 102 (5.4) | 212 (11.0) | | 88 (4.4) | 141 (7.4) | 7 (0.3) | 31 (1.6) | 870 (5.2) |
| Family dependent | 482 (16.7) | 411 (20.6) | 530 (28.2) | 389 (20.3) | | 847 (42.5) | 275 (14.4) | 189 (9.0) | 418 (21.4) | 3541 (21.3) |
| Private | 173 (6.0) | 100 (5.0) | 48 (2.6) | 83 (4.3) | | 59 (3.0) | 119 (6.2) | 739 (35.2) | 281 (14.4) | 1602 (9.6) |
| **Living alone** | 257 (8.9) | 254 (12.7) | 89 (4.7) | 61 (3.1) | | 218 (10.9) | 461 (24.1) | 101 (4.8) | 161 (8.2) | 1602 (9.6) |
| **Care Dependency – MV** | 348 | 4 | 2 | 2 | | 0 | 7 | 0 | 18 | 381 |
| Yes | 131 (5.1) | 135 (6.8) | 53 (2.8) | 84 (4.3) | | 80 (4.0) | 199 (10.4) | 116 (5.5) | 89 (4.6) | 1433 (8.8) |
| **No. of physical impairments- MV^*^** | 6 | 2 | 2 | 23 | | 0 | 5 | 0 | 0 | 38 |
| None | 1272 (44.0) | 595 (29.8) | 871 (46.3) | 747 (38.9) | | 831 (41.7) | 694 (36.3) | 1040 (49.5) | 970 (49.7) | 7020 (42.2) |
| One or two | 1334 (46.1) | 942 (47.1) | 757 (40.2) | 690 (35.9) | | 821 (41.2) | 834 (43.7) | 830 (39.5) | 801 (41.0) | 7009 (42.1) |
| Three or more | 285 (9.9) | 461 (23.1) | 254 (13.5) | 484 (25.2) | | 340 (17.1) | 381 (20.0) | 231 (11.0) | 182 (9.3) | 2618 (15.7) |
| **Depression** | 143 (4.9) | 277 (13.9) | 103 (5.5) | 106 (5.5) | | 92 (4.6) | 47 (2.5) | 10 (0.5) | 165 (8.5) | 943 (5.7) |
| **Dementia - MV** | 0 | 0 | 0 | 0 | | 0 | 9 | 0 | 0 | 9 |
| Yes | 281 (9.7) | 233 (11.7) | 122 (6.5) | 131 (6.7) | | 173 (8.7) | 145 (7.6) | 91 (4.3) | 155 (7.9) | 1331 (8.0) |

^*^ *MV: missing value; Data are N and % unless otherwise stated.*
