# Supplementary material for: Loneliness Among Older Adults in Latin America, China, and India: Prevalence, Correlates and Association With Mortality
Source: Int J Public Health. 2021 Mar 31;66:604449. doi: 10.3389/ijph.2021.604449 (PMC8565277; doi:10.3389/ijph.2021.604449)
Supplement: Supplementary file 2 [file Table2.docx]

**Supplementary File 2 Correlates of loneliness with adjusted prevalence ratios (robust 95% confidence interval) in Latin America, China, and India (2003-2007)**

| **Characteristics** | **Cuba** | **Dominican Republic** | **Peru urban** | **Peru rural** | **Venezuela** | **Mexico urban** | **Mexico rural** | **Puerto Rico** | **China urban** | **India urban** | **India rural** |
| --- | --- | --- | --- | --- | --- | --- | --- | --- | --- | --- | --- |
| **Age** (65-110 years old) | 0.99 (0.98-1.00) | 1.00 (1.00-1.01) | 0.99 (0.98-1.01) | 0.98 (0.96-1.01) | 0.99 (0.98-1.00) | 1.01 (0.99-1.02) | 1.01 (1.00-1.02) | 0.98 (0.97-0.99) | 1.00 (0.95-1.05) | 0.99 (0.97-1.01) | 1.01 (0.99-1.03) |
| **Gender** Male (ref. female) | 0.77 (0.64-0.92) | 1.02 (0.88-1.19) | 0.75 (0.62-0.92) | 0.79 (0.56-1.11) | 0.84 (0.69-1.03) | 0.88 (0.70-1.10) | 0.75 (0.63-0.90) | 0.76 (0.64-0.91) | 1.06 (0.59-1.92) | 0.96 (0.71-1.29) | 0.82 (0.63-1.07) |
| **Higher Education** (ref. lower) | 0.98 (0.91-1.04) | 1.00 (0.93-1.07) | 0.95 (0.87-1.02) | 0.90 (0.74-1.08) | 1.01 (0.92-1.10) | 0.95 (0.87-1.04) | 0.87 (0.79-0.97) | 0.96 (0.90-1.02) | 0.89 (0.71-1.12) | 0.82 (0.71-0.94) | 0.78 (0.66-0.93) |
| **Household Assets** (ref. fewer) | 0.96 (0.89-1.03) | 0.89 (0.83-0.95) | 0.94 (0.86-1.03) | 0.99 (0.84-1.17) | 0.93 (0.85-1.02) | 0.90 (0.83-0.99) | 0.89 (0.82-0.96) | 1.01 (0.93-1.09) | 1.39 (0.96-2.02) | 0.75 (0.65-0.86) | 0.82 (0.75-0.90) |
| **Pension** (ref. none) | 0.86 (0.72-1.02) | 0.92 (0.80-1.06) | 0.97 (0.83-1.13) | 0.82 (0.59-1.13) | 0.92 (0.79-1.08) | 0.84 (0.68-1.03) | 0.95 (0.82-1.12) | 1.12 (0.98-1.28) | 0.75 (0.32-1.72) | 0.92 (0.57-1.47) | 0.88 (0.74-1.06) |
| **Marital Status (**ref. never married) | |  |  |  |  |  |  |  |  |  |  |
| Married/ Cohabiting | 0.68 (0.53-0.88) | 0.81 (0.59-1.11) | 0.99 (0.74-1.32) | 1.16 (0.64-2.11) | 0.75 (0.55-1.02) | 0.94 (0.60-1.49) | 0.67 (0.48-0.94) | 0.86 (0.62-1.18) | n/a* | 0.54 (0.26-1.15) | 0.59 (0.19-1.84) |
| Widowed | 1.18 (0.93-1.50) | 1.30 (0.97-1.74) | 1.48 (1.12-1.97) | 1.65 (0.89-3.05) | 1.52 (1.15-2.01) | 1.35 (0.86-2.10) | 0.94 (0.68-1.31) | 1.46 (1.06-2.00) | n/a | 0.71 (0.34-1.51) | 0.55 (0.18-1.74) |
| Divorced/ Separated | 1.25 (0.98-1.61) | 1.33 (1.00-1.78) | 1.33 (0.92-1.93) | 2.53 (1.17-5.48) | 1.18 (0.86-1.61) | 1.43 (0.88-2.32) | 0.98 (0.67-1.44) | 1.34 (0.97-1.87) | n/a | 0.77 (0.32-1.85) | 0.68 (0.21-2.24) |
| **Social network** (ref. locally integrated) | |  |  |  |  |  |  |  |  |  |  |
| Locally self-contained | 1.15 (0.88-1.50) | 1.29 (1.04-1.59) | 1.45 (1.07-1.97) | 1.36 (0.80-2.31) | 1.30 (1.01-1.67) | 1.17 (0.74-1.87) | 1.30 (0.99-1.70) | 1.29 (1.06-1.57) | 0.25 (0.10-0.61) | 0.89 (0.49-1.64) | 1.10 (0.75-1.62) |
| Wider community-focused | 1.27 (0.89-1.81) | 1.21 (0.99-1.48) | 1.59 (1.19-2.13) | 1.04 (0.58-1.86) | 1.11 (0.87-1.41) | 0.95 (0.58-1.55) | 0.86 (0.55-1.36) | 1.34 (1.09-1.66) | 0.00 (0.00-0.00) | 0.39 (0.10-1.46) | 1.31 (0.39-4.40) |
| Family dependent | 0.97 (0.80-1.18) | 0.96 (0.81-1.13) | 1.17 (0.99-1.39) | 1.09 (0.74-1.60) | 1.14 (0.94-1.38) | 1.17 (0.96-1.42) | 0.83 (0.70-0.97) | 1.13 (0.93-1.37) | 0.05 (0.01-0.21) | 1.13 (0.84-1.52) | 1.62 (1.31-2.00) |
| Private | 1.26 (1.00-1.59) | 1.22 (0.95-1.57) | 1.08 (0.70-1.66) | 0.49 (0.07-3.47) | 0.68 (0.43-1.07) | 1.59 (1.11-2.28) | 1.08 (0.62-1.90) | 1.33 (1.04-1.70) | 0.29 (0.16-0.55) | 0.98 (0.70-1.38) | 1.17 (0.84-1.61) |
| **Living alone** (ref. living with others) | 1.62 (1.35-1.94) | 1.35 (1.14-1.59) | 1.12 (0.83-1.53) | 1.67 (1.04-2.69) | 0.98 (0.68-1.42) | 1.32 (1.03-1.69) | 1.22 (0.99-1.49) | 1.47 (1.23-1.74) | 0.92 (0.25-3.35) | 1.69 (1.12-2.56) | 1.62 (1.31-2.00) |
| **Physical impairments** (ref. less) | 1.41 (1.27-1.55) | 1.26 (1.15-1.39) | 1.39 (1.26-1.54) | 1.28 (1.03-1.60) | 1.27 (1.15-1.41) | 1.30 (1.15-1.48) | 1.27 (1.15-1.41) | 1.39 (1.27-1.53) | 1.73 (1.17-2.56) | 1.07 (0.86-1.33) | 1.21 (1.07-1.37) |
| **Dependence** (ref. no needs for much care) | 1.01 (0.73-1.39) | 1.26 (1.03-1.53) | 0.79 (0.51-1.23) | 1.02 (0.32-3.22) | 1.28 (0.92-1.78) | 1.12 (0.82-1.53) | 0.72 (0.45-1.17) | 1.21 (0.94-1.57) | 1.36 (0.50-3.71) | 1.64 (0.80-3.38) | 1.39 (0.72-2.71) |
| **Depression** (ref. non-case) | 2.29 (1.91-2.74) | 1.87 (1.63-2.14) | 1.77 (1.49-2.11) | 2.22 (1.26-3.91) | 1.84 (1.49-2.26) | 1.75 (1.38-2.21) | 1.99 (1.69-2.36) | 1.86 (1.51-2.30) | 10.87 (3.02-39.08) | 2.37 (1.74-3.22) | 1.79 (1.49-2.14) |
| **Dementia** (ref. non-case) | 0.99 (0.77-1.28) | 1.01 (0.83-1.23) | 0.83 (0.58-1.19) | 0.91 (0.46-1.83) | 1.30 (1.00-1.68) | 1.36 (1.05-1.77) | 1.04 (0.84-1.29) | 1.15 (0.90-1.47) | 1.05 (0.28-3.97) | 0.88 (0.57-1.35) | 0.89 (0.69-1.15) |

***** Estimates could not be obtained due to too few exposed in never married/ divorced/ separated categories for China;

** Results for China rural was removed because lack of power.
