# Supplementary material for: Loneliness Among Older Adults in Latin America, China, and India: Prevalence, Correlates and Association With Mortality
Source: Int J Public Health. 2021 Mar 31;66:604449. doi: 10.3389/ijph.2021.604449 (PMC8565277; doi:10.3389/ijph.2021.604449)
Supplement: Supplementary file 3 [file Image1.pdf]

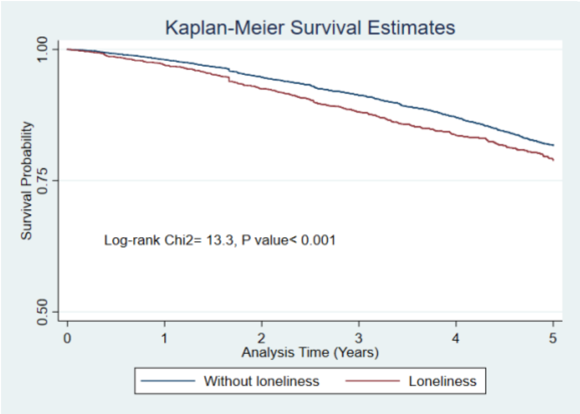

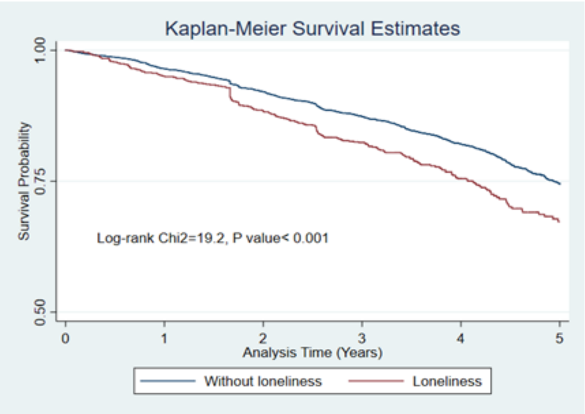

(A) Female

(B) Male

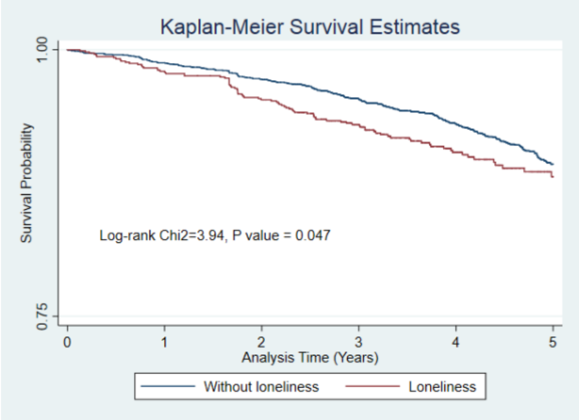

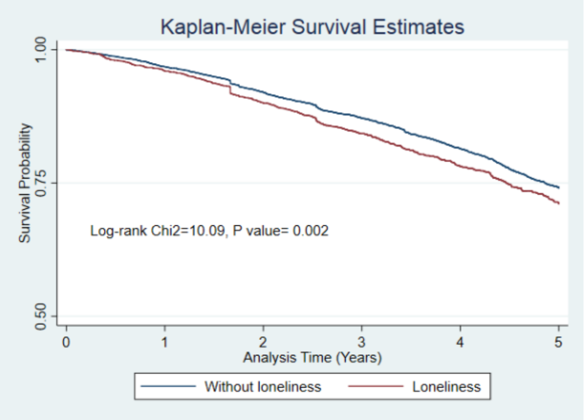

(C) Lower than 70 years old

(D) 70 years old and above

Supplementary File 3 Crude Kaplan-Meier survival curves for 5-year all-cause mortality, stratified by self-reported loneliness and gender (A) Female (B) Male; by age (C) Lower than 70 years old (D) 70 years old and above
