# Supplementary material for: Loneliness Among Older Adults in Latin America, China, and India: Prevalence, Correlates and Association With Mortality
Source: Int J Public Health. 2021 Mar 31;66:604449. doi: 10.3389/ijph.2021.604449 (PMC8565277; doi:10.3389/ijph.2021.604449)
Supplement: Supplementary file 4 [file Image2.pdf]

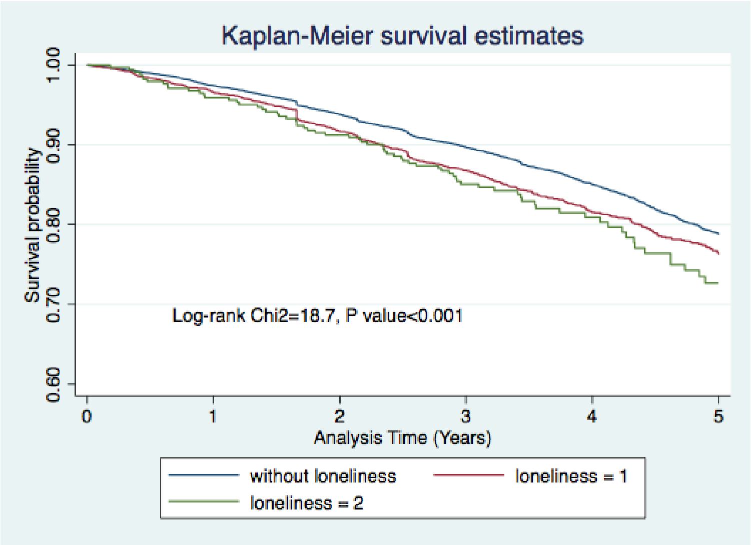

*** "without depression"/ loneliness 1 "with loneliness, but mild to moderate intensity, infrequent or fleeting"/ Loneliness 2 "with loneliness, and severe, frequent or persistent"**

Supplementary File 4 Crude Kaplan-Meier survival curves for 5-year all-cause mortality
